# Risks and Management of Glucocorticoid Therapy for Patients with Rheumatic Disease Having Surgery

**DOI:** 10.1007/s11926-026-01221-3

**Published:** 2026-06-04

**Authors:** Kunjam Modha, Christopher Whinney

**Affiliations:** Department of Hospital Medicine, Cleveland Clinic Foundation, 9500 Euclid Avenue, Cleveland, OH 44195 USA

**Keywords:** Glucocorticoids, Perioperative, Rheumatic disease, Stress steroids

## Abstract

**Purpose of Review:**

The purpose of this review was to identify and characterize the diverse risks and benefits of glucocorticoid use in the perioperative period in patients with rheumatic diseases.

**Recent Findings:**

Glucocorticoids increase the risk of surgical site infection, wound dehiscence, pneumonia, unplanned intubation and readmissions. The risk of perioperative adrenal insufficiency is rare. Guidelines specifically for knee and hip arthroplasties do not recommend stress dosing of steroids.

**Summary:**

Continuation of home doses of glucocorticoids is adequate for most patients. Stress dosing of glucocorticoids should be reserved for patients with biochemically confirmed adrenal dysfunction or are receiving supraphysiologic doses of glucocorticoids going for moderate to high-risk surgeries.

## Introduction

Chronic glucocorticoids (GC) are the mainstay of treatment of various rheumatological diseases. Patients with rheumatic disorders are at a higher risk of complications around surgery as a result of the underlying disease itself as well as the therapies involved. The very anti-inflammatory effects of glucocorticoids that help in disease control can also poorly affect surgical outcomes.

## Perioperative Implications of Glucocorticoid Use

Corticosteroids are known to downregulate cytokines, decrease fibroblast proliferation and negatively affect collagen modeling raising concerns for increased risk of postoperative complications especially wound healing. A retrospective analysis of data on 403,566 patients collected by the American College of Surgeons National Surgical Quality Improvement Program reported on the risk of postoperative complications in patients who had undergone total hip and knee arthroplasties. 14,774 patients were prescribed chronic GCs [1]. A statistically significant difference was observed in perioperative complications in patients prescribed GCs that included higher rates of surgical site infection, deep incisional surgical site infection, wound dehiscence, pneumonia, unplanned intubation, urinary tract infection, and readmission. The authors did note however, that there were baseline differences in patient cohorts with regards to cardiac, pulmonary and dialysis dependency as well as obesity and American Society of Anesthesiology class. This likely reflects corticosteroid use in a sicker population in general and poorer outcomes due to contribution of several factors. Another retrospective study of 432 patients with rheumatoid arthritis undergoing knee and hip arthroplasties included 30% patients on chronic GC therapy and 90% of patients also received supraphysiologic doses of GC perioperatively [2]. The authors found that a higher cumulative GC dose was associated with a higher incidence of hyperglycemia, surgical site infections, catheter-associated urinary tract infections, prosthetic joint infections, sepsis and deep venous thrombosis. For every 10 mg increase in cumulative GC dose (prednisone equivalent), there was an 8.4% increase in the odds of a patient experiencing an early postoperative complication. Chronic home GC dose was not associated with increased risk of any complication.

On the other hand, chronic GC therapy may be crucial for maintenance of disease control perioperatively. A recent retrospective analysis of 337 patients undergoing total hip arthroplasty reported a significant increase in rates of dislocation, infection, periprosthetic fracture and aseptic loosening with increase in disease activity [3].

Thus, it is essential to identify patients who will benefit the most from “stress dose steroids” and for who, the benefits of this strategy will likely outweigh the risks. Stress dosing of steroids is defined as an augmentation of the home steroid dose in response to the surgical stress to mitigate the risk of perioperative adrenal crisis.

## Secondary Adrenal Insufficiency and Surgery

Intact adrenal function is crucial to the surgical environment in order to maintain hemodynamic stability and end organ perfusion. The stress of surgery, trauma, and critical illness, and the vasodilation associated with anesthesia, in conjunction with blood loss and fluid shifts, could theoretically lead to hypotension and shock in the setting of inadequate adrenal reserves [4]. While this is likely the case in patients with confirmed adrenal insufficiency on true adrenal replacement therapy, it is less clear in patients receiving exogenous corticosteroids for other therapeutic purposes.

Suppression of the HPA axis with exogenous corticosteroids is quite variable depending on dose, frequency, and patient-related factors including age, weight, hepatic function, concomitant drugs metabolized by the P450 system, and concurrent illnesses. A diminished response to ACTH stimulation testing is reported to last for 5 days after a similar duration of 25 mg prednisone therapy [5]. Corticosteroid dose, frequency (i.e., daily or alternate day dosing), and duration of therapy are key historical elements to help inform decision-making. Physical examination is often limited in utility, but may demonstrate Cushingoid facies, abdominal and flank striae, and centripetal obesity as manifestations or glucocorticoid excess from exogenous supplementation. Some experts advocate for morning cortisol assays and/or ACTH stimulation testing to assess the hypothalamic-pituitary-adrenal (HPA) axis; however, the interpretation of this testing is limited with the use of exogenous corticosteroids; in addition, there have been no studies to correlate the results of this testing with intraoperative or postoperative adrenal crisis [6, 7].

Limited high quality evidence exists supporting the practice of perioperative high dose glucocorticoids. The use of stress dose steroids before surgery stems from case reports from the 1950s of patients who developed circulatory shock after surgery associated with withdrawal of their usual cortisone therapy [8, 9]. A review by Salem and colleagues found that most replacement doses of glucocorticoids were likely excessive and that continuing the patient on their current dose of corticosteroid through surgery is appropriate [10]. A systematic review of two randomized controlled trials and seven cohort studies found no evidence of adrenal crisis except for two patients in the cohort studies who had their usual doses stopped prior to surgery and developed unexplained hypotension [6]. Guidelines published in the United Kingdom in 2020 suggest 100 mg of hydrocortisone for moderate-risk procedures [7]; however, the American College of Rheumatology/American Association of Hip and Knee Surgeons guidelines from 2022 maintain the recommendation from 2017 of continuing current daily dose of glucocorticoids for hip and knee replacements rather than administering supraphysiologic doses of glucocorticoids (conditional recommendation) [11]. In addition, it is now a recommendation in anesthesiology guidelines to administer dexamethasone in the perioperative period to mitigate the inflammatory cascade, reduce pain, and preventively address nausea and vomiting, so this may make glucocorticoid replacement decisions less relevant in most cases [12]. The one exception to this scenario is patients with confirmed physiologic adrenal insufficiency, as dexamethasone has no mineralocorticoid activity; these patients should have mineralocorticoid replacement through the perioperative period.

## Administration of Stress Dose Glucocorticoids

When considering providing supplemental glucocorticoids around surgery, clarify if they have documented adrenal insufficiency or have a clinical suspicion of tertiary insufficiency from exogenous glucocorticoids [13]. Identify any prior history of adrenalectomy, tuberculosis, sarcoid, or other etiology, and investigate any prior laboratory assessments including morning cortisol determinations and/or ACTH stimulation testing. Corticosteroid dose, frequency (i.e., daily or alternate day dosing), and duration of therapy are key historical elements to help inform decision-making. Physical examination may demonstrate Cushingoid facies, abdominal and flank striae, and centripetal obesity as manifestations of glucocorticoid excess from exogenous supplementation. The risk for HPA axis suppression from chronic glucocorticoid therapy is variable, but is related to the type of glucocorticoid, systemic distribution, dose, and duration of treatment. Systemic versus inhaled or topical therapy, epidural or intra-articular injections, long-acting agents, higher doses, longer durations of therapy, higher potency, nighttime administration, multiple treatment cycles, and multiple daily doses all contribute to risk for HPA axis suppression.

A low morning cortisol level and/or a positive ACTH stimulation test helps to confirm the diagnosis. This is defined as an increase in serum cortisol by less than 7 mg/dl or an absolute cortisol level less than 18 ^mg^/dl 1 h after the administration of 1 mcg ACTH. This does not need to be repeated in the perioperative setting unless the diagnosis is in question or if the patient is already taking exogenous corticosteroids. Other experts have suggested the use of ACTH stimulation tests for risk stratification, but there have been no studies to correlate the results of this testing with intraoperative or postoperative adrenal crisis, so we do not recommend conducting repeat testing, unless it is indicated outside of the perioperative milieu.

There are numerous recommendations in the literature as to how to supplement glucocorticoids to “cover” patients for perioperative adrenal crisis avoidance [13]. Many experts believe that we are likely providing an excessive amount of glucocorticoid coverage, and more recent trends have been toward lower doses of glucocorticoids. Arafah found that patients administered 25 mg of intravenous hydrocortisone every 6 h for 24 h, followed by 15 mg every 6 h for 24 h before surgery resulted in no adverse events or evidence of adrenal crisis, and demonstrated nadir cortisol levels were in the range of values or exceeded those noted in patients without adrenal dysfunction [14]. Chen Cardenas and colleagues provide recommendations for replacement doses of glucocorticoids in patients currently taking them on the basis of surgical stress, in patients who have stopped or plan to stop them before surgery, and in patients in adrenal crisis [13]. (Table 1). Prolonged tapering doses are not warranted in this scenario; once the patient is able to tolerate oral intake by postoperative day two or three, resuming the patient’s home glucocorticoid regimen is sufficient for adrenal coverage.

**Table 1:** Suggested perioperative regimens for glucocorticoid induced adrenal insufficiency

Glucocorticoid use	Degree of surgical stress	Suggested glucocorticoid regimen
Patients currently on glucocorticoids	Minor (skin or subcutaneous procedures, inguinal hernia repair, breast biopsy, endoscopic, minor gynecology and otolaryngology procedures); estimated blood loss < 500mL)	• Continue daily dose of glucocorticoid • 25 mg IV hydrocortisone at induction if unable to take orally • Resume oral daily preoperative glucocorticoid regimen
	Moderate (open or laparoscopic digestive tract procedures, cholecystectomy, thyroidectomy, genitourinary organ removal, joint replacement, laminectomy); estimated blood loss 500-1500mL	• Continue daily dose of glucocorticoid • 25–50 mg of hydrocortisone IV at induction • 15–25 mg hydrocortisone every 6 h until oral intake is tolerated and hemodynamically stable • Resume oral daily preoperative glucocorticoid regimen
	Major (large orthopedic-spinal, oropharyngeal, or genitourinary repair or reconstruction; intracranial, major vascular or cardiothoracic procedure); estimated blood loss > 1500mL and/or anticipated postoperative ICU stay	• Continue daily dose of glucocorticoid • 50 mg of hydrocortisone IV at induction • 25 mg of hydrocortisone IV every 6 h on day 1 and until hemodynamically stable, then 15 mg IV every 6 h until oral intake is tolerated • Resume oral daily preoperative glucocorticoid regimen
Patients who have stopped or plan to stop glucocorticoids before surgery		• Assess HPA axis in appropriate patients • The closer the date of discontinuing glucocorticoids before surgery, the higher the risk of adrenal insufficiency • Treat based on the degree of surgical stress in those who have abnormal HPA axis
Adrenal Crisis		• 100 mg of hydrocortisone IV (IM if no IV access) • 50 mg every 6 h until hemodynamically stable and then taper • Taper depending on clinical response-intravenous fluids (normal saline), dextrose 5% if hypoglycemia

Adapted from Chen Cardenas et al.^13^*A*bbreviations*: IV *intravenous,* ICU *intensive care uni*t*,* HPA *Hypothalamic pituitary axis,* IM *intramuscular

## Conclusion

In summary, there are risks and benefits of perioperative use of glucocorticoids. Patients with proven adrenal insufficiency should be administered glucocorticoid and mineralocorticoid replacement doses perioperatively. In patients with secondary/tertiary adrenal insufficiency, a detailed history about current/prior glucocorticoid dosing should be obtained in addition to a comprehensive physical exam. ACTH testing can be supplemented but is not mandatory. Since higher cumulative GC dose is associated with perioperative complications, ideally, patients should have their chronic glucocorticoid dose lowered to the safest dose possible to control their disease activity. When a decision to prescribe stress dose steroids is made, degree of surgical stress should guide dosing. For total knee and hip arthroplasties specifically, guidelines recommend continuing normal daily glucocorticoid dosing without supraphysiologic replacement doses.

## Key References

- Chukir, T., Goodman, S.M., Tornberg, H., Do, H., Thomas, C., Sigmund, A., Sculco, P., Figgie, M., Mehta, B., Russell, L. and Stein, E. (2021), Perioperative Glucocorticoids in Patients With Rheumatoid Arthritis Having Total Joint Replacements: Help or Harm? ACR Open Rheumatology, 3: 654-659
  - ○ This study evaluates the risks and benefits of perioperative glucocorticoid use in rheumatoid arthritis patients undergoing total joint replacement. While glucocorticoids may reduce inflammatory complications, the authors highlight potential increases in postoperative infection and wound-healing risk.
- Lai Y, Tang H, Ding Z, Huang C, Cai Y, Luo Z, Zhou Z. Association between disease activity of rheumatoid arthritis and risk of complications following total hip arthroplasty: a retrospective cohort study. J Orthop Surg Res. 2024 Jul 31;19(1):455. PMID: 39085960
  - ○ This retrospective cohort study shows that higher rheumatoid arthritis disease activity is associated with increased postoperative complications after total hip arthroplasty. Although limited by its observational design and potential confounding, it highlights disease activity as an important, potentially modifiable preoperative risk factor.
- Chen Cardenas SM, Santhanam, P, Morris-Wiseman L, et al. Perioperative Evaluation and Management of Patients on Glucocorticoids. J Endo Soc 2023; 7: 1-6. PMID: 3654564
  - ○ This consensus-based article provides practical guidance relevant to surgical patients with chronic steroid exposure. It emphasizes risk stratification and selective use of stress-dose steroids rather than routine supplementation.

## Data Availability

No datasets were generated or analysed during the current study.
